# Disorders Associated With Diverse, Recurrent Deletions and Duplications at 1q21.1

**DOI:** 10.3389/fgene.2020.00577

**Published:** 2020-06-23

**Authors:** Hui Pang, Xiaowei Yu, Young Mi Kim, Xianfu Wang, Jeremy K. Jinkins, Jianing Yin, Shibo Li, Hongcang Gu

**Affiliations:** ^1^Department of Pediatrics, The University of Oklahoma Health Sciences Center, Oklahoma, OK, United States; ^2^The First Affiliated Hospital of Jilin University, Changchun, China; ^3^Broad Institute of MIT and Harvard, Cambridge, MA, United States

**Keywords:** 1q21.1 deletion, 1q21.1 duplication, TAR deletion, proximal 1q21.1 duplication, non-allelic homologous recombination, segmental duplication, breakpoints

## Abstract

The subchromosomal region 1q21.1 is one of the hotspots in the human genome for deletions and reciprocal duplications, owing to the existence of hundreds of segmental duplications. Recurrent deletions and duplications in this region are thought to be causative in patients with variable clinical manifestations. Based on the genomic locations, deletions and duplications at the 1q21.1 locus have been associated with distinguishable syndromes: chromosome 1q21.1 deletion syndrome, chromosome 1q21.1 duplication syndrome, and thrombocytopenia-absent radius (TAR) syndrome, which is partially due to deletions at the proximal 1q21.1 region. We report here diverse, recurrent deletions and duplications at the 1q21.1 locus in 36 patients from a cohort of 5,200 individuals. Among the 36 patients, 18 patients carry 1q21.1 deletions, nine individuals have reciprocal duplications at 1q21.1, two patients share an identical short deletion, and the remaining seven possess variable sizes of duplications at the proximal 1q21.1 region. Furthermore, we provide cytogenetic characterization and detailed clinical features for each patient. Notably, duplications at the proximal 1q21.1 region have not been associated with a defined disorder in publications. However, recurrent duplications at the proximal 1q21.1 region among the seven patients strongly suggested that the variants are likely pathogenic. The common phenotypical features of those disorders are also summarized to facilitate clinical diagnoses and genetic counseling.

## Introduction

Recurrent deletions and duplications at the subchromosomal region 1q21.1 (GRCh37/hg19, chr1:144.0–149.5 Mb) have been reported in patients with diverse clinical features (Brunetti-Pierri et al., 2008; Mefford et al., 2008; Cooper et al., 2011; Girirajan et al., 2012). The mechanism of chromosomal deletion and duplication is most likely due to segmental duplications (a.k.a., low copy repeats) in the region (Brunetti-Pierri et al., 2008). These low copy repeats cluster together and form four segmental duplication blocks with a size range of 270 kb–2.2 Mb, making this subchromosomal region a hotspot for non-allelic homologous recombination (NAHR) (Mefford et al., 2008; Cooper et al., 2011; Girirajan et al., 2012). Not surprisingly, chromosomal breakpoints (BPs) in patients with 1q21.1deletions and duplications have been exclusively mapped to the four segmental duplication blocks, designated as BP1–BP4 according to the orientation from centromere to telomere (Brunetti-Pierri et al., 2008; Rosenfeld et al., 2012).

As hundreds of individuals possessing the 1q21.1 deletions or the reciprocal duplications manifest phenotypic abnormalities, documentation and categorization of three syndromes have been included in the Online Mendelian Inheritance in Man (OMIM). Those rare disorders include chromosome 1q21.1 deletion syndrome (MIM 612474), chromosome 1q21.1 duplication syndrome (MIM 612475), and TAR syndrome (MIM 274000). Chromosome 1q21.1 deletion and duplication syndromes share certain clinical phenotypes such as developmental delays, craniofacial abnormalities, and cardiac anomalies. Other relative common phenotypes include intellectual disabilities (IDs), attention deficit hyperactive disorders, autism spectrum disorder, and schizophrenia (Brunetti-Pierri et al., 2008; Mefford et al., 2008; Harvard et al., 2011; Van Dijck et al., 2015; Verhagen et al., 2015; Bernier et al., 2016; Buse et al., 2017). Contrastingly, patients with 1q21.1 deletion syndrome are more likely to display features of microcephaly and/or schizophrenia, whereas carriers of 1q21.1 duplications are inclined to present symptoms of macrocephaly and/or autism (Mefford et al., 2008; Levinson et al., 2011; Crespi and Crofts, 2012; Girirajan et al., 2012; Dolcetti et al., 2013). Based on the molecular sizes of CNVs, 1q21.1 deletions and duplications have been grouped into two classes (Brunetti-Pierri et al., 2008). The class I deletions and reciprocal duplications typically occur at the 1q21.1 distal region between BP3 and BP4 with a size range of 800 kb–2 Mb, and the class II deletions and reciprocal duplications commonly reside between BP1/BP2 and BP4 with relatively bigger sizes of ∼3 Mb or larger (Brunetti-Pierri et al., 2008; Cooper et al., 2011).

Thrombocytopenia-absent radius syndrome was initially reported in the 1950s, with the diagnostic criteria for this syndrome defined by Hall et al. in 1969 (Gross et al., 1956; Shaw and Oliver, 1959; Hall et al., 1969). The characteristic features of TAR syndrome are bilateral absence of radial bones in the forearms and thrombocytopenia (<50 platelets/nl) (Albers et al., 2013). Less common symptoms include hand and foot abnormalities, congenital heart defects, renal anomalies, and cow milk intolerance (Greenhalgh et al., 2002; Rosenfeld et al., 2012; Albers et al., 2013). Nevertheless, it was only until recently that the genetic basis and the inheritance pattern of TAR syndrome were ascertained (Klopocki et al., 2007). Most patients diagnosed with TAR syndrome have compound heterozygous mutations, including a deletion at the proximal 1q21.1 region (a.k.a., TAR deletion), with a minimal size of 200 kb that encompasses the RBM8A gene and a point mutation in the non-coding region of RBM8A (Albers et al., 2013; Toriello et al., 2016). Remarkably, the reciprocal duplication of the TAR deletion region, also being called proximal 1q21.1 duplication, is significantly enriched in patients with variable clinical features when compared to control groups in the two large cohort studies (Cooper et al., 2011; Rosenfeld et al., 2012). In one report, Rosenfeld et al. (2012) proposed that the proximal 1q21.1 duplications were causative to the patients’ phenotypical features. However, more supportive evidence is needed to refine their roles in the symptoms manifested in the patients.

To better understand the disorders associated with deletions and duplications at the 1q21.1 locus, we investigated more than 5,200 clinical tests conducted in the last 10 years at our laboratory that has been accredited by the College of American Pathologist (CAP) since 2000. Our investigation resulted in 36 cases with one causative or likely causative variant at the 1q21.1 locus. Molecular characterizations and clinical manifestations are described.

## Materials and Methods

### Patients and Sample Collections

Most of the patients were from the state of Oklahoma, which has a racial composition as follows: 72.0% White, 8.7% Native American, 7.4% African American, 1.7% Asian, and 10.2% of other races according to the 2000 census data. Three patients were from China, and the samples were provided by our collaborator. The peripheral blood samples were collected from patients and their parents. This study was approved by the ethical committee of the First Hospital of Jilin University and by the Institutional Review Board of the University of Oklahoma Health Sciences Center, respectively. Informed consent forms were signed by the patients or their guardians, in compliance with the Declaration of Helsinki.

### Microarray Tests and Karyotype Analysis

Genomic DNA was isolated from the peripheral blood samples using the Maxwell RSC Blood DNA kit (Promega) as per the manufacturer’s instructions. Comparative genomic hybridization (CGH) assays were conducted with the SurePrint HD array (2 × 400 K V.1.0, Agilent Inc.), which was designed to detect CNVs across the whole genome. Of note is that the array contained 400 K oligonucleotides with a median probe spanning of ∼5.3 kb that represented the coding and the non-coding sequences in the human genome. The DNA samples from healthy individuals were used as controls for data analysis. We analyzed the CGH array data using the CytoGenomics software provided by Agilent.

Whole-genome single nucleotide polymorphism (SNP) array tests were executed by utilizing the Infinium CytoSNP-850K kit (v1.1 BeadChip, Illumina), following the manufacturer’s guidelines. Specifically, the Illumina chip contained nearly 850,000 empirically selected SNPs spanning the whole genome. The average inter-probe distance was approximately 1.8 kb, and the overall effective resolution was around 18 kb. The processed chip was scanned on the NextSeq550 system (Illumina). The data were analyzed using the BlueFuse Multi v4.3 software (Illumina).

Cell preparation and karyotype analysis were performed as previously reported (Kim et al., 2011). For each patient, 20 metaphase cells were analyzed by G-banding.

### Targeted Sequencing and Whole-Exome Sequencing

The customized panel for oral–facial–digital (OFD) syndrome included five genes: C2CD3, CPLANE1, DDX59, OFD1, and TCTN3. Baits for the OFD gene panel and whole-exome sequencing (WES), which targeted on comprehensive medical exomes, were purchased from Agilent. Both the targeted sequencing library and the WES library for patient 33 were constructed using the Agilent SureSelect clinical research exome kit, except that the hybrid captures were performed using different baits as described above. Sequencing was conducted on a NextSeq550 sequencer (Illumina) with 100 bp paired-end reads. The DNA sequence was aligned to the human reference genome (UCSC hg19). We built a sequence processing pipeline for analyzing both the targeted gene panel and the WES data by incorporating two commercial software, CLC biomedical genomic workbench from QIAGEN and AlamutBatch from Interactive Biosoftware. The evaluation and the classification of variants were performed following the American College of Medical Genetics (ACMG) recommendations (Richards et al., 2015).

## Results

### Molecular Characterizations of CNVs at 1q21.1

We analyzed 5,200 microarray tests predominantly for pediatric patients with developmental delays, IDs, craniofacial abnormalities, congenital heart defects, and other abnormalities. Our investigation identified 38 individuals carrying either one copy of deletion or duplication at the 1q21.1 locus. Of note is that one patient had a 3.0-Mb deletion at 1q21.1 and an additional pathogenic deletion of ∼1.1 Mb at 17p13.2 (chr17:5,024,275–6,074,275). Another patient carried a proximal 1q21.1 duplication of ∼436 kb and an extra, likely pathogenic, deletion at 19p13.3 (chr19:259,395–1,045,363). After excluding those two cases, 36 individuals with either a deletion or a duplication at the 1q21.1 locus were subjected to further examinations. Notably, case 22 also carried one 1.1-Mb duplication at 13q21.33, and case 23 had a 409-kb deletion at 15q21.2. However, both the duplication at 13q21.33 and the deletion of 15q21.2 have not been reported as pathogenic in any patient with a disorder. In addition, the chromosomal analysis for 10 out of 36 cases all showed a normal karyotype. Our final subjects for systemic analyses included 31 probands with variable clinical manifestations and five individuals showing unnoticeable or very mild symptoms (Supplementary Table S1).

**FIGURE 1:**
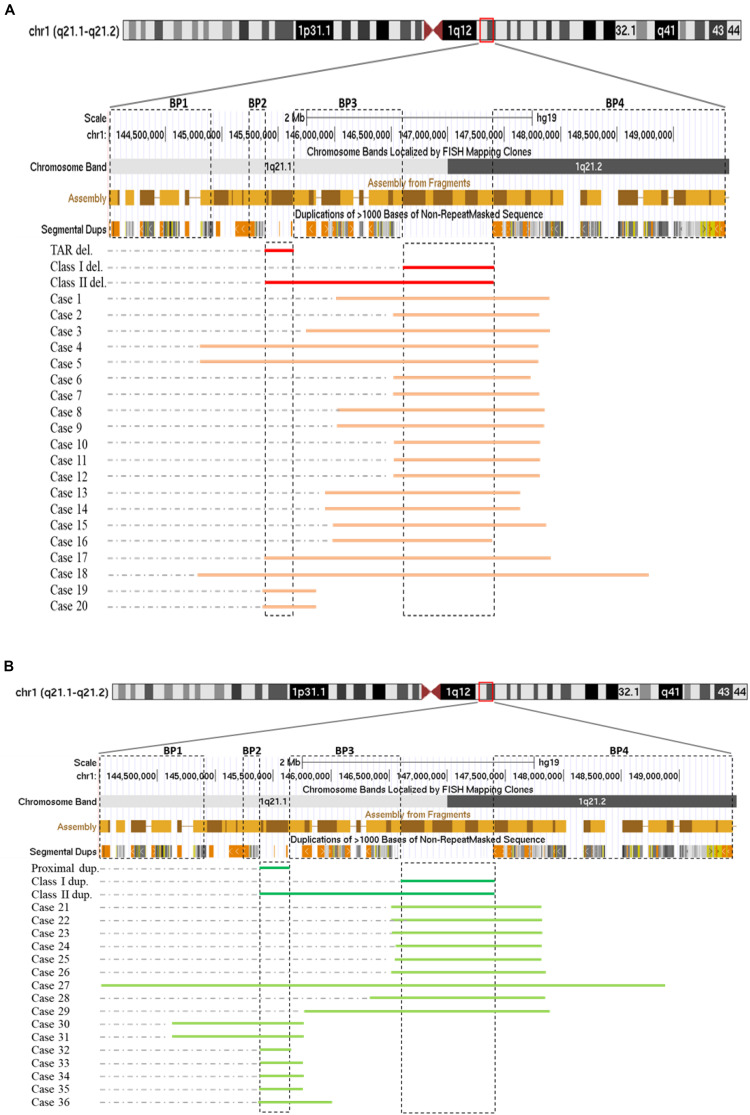
Schematic representations of deletions **(A)** and duplications **(B)** at 1q21.1 (GRCh37/hg19, 144.0–149.5 Mb). Breakpoint regions (BP1–BP4) in dotted squares were delineated according to Mefford et al. (2008). Class I, class II, and thrombocytopenia-absent radius (TAR) deletions (labeled as class I del, class II del, and TAR del) were drawn based on Brunetti-Pierri et al. (2008) with modification. In a similar way, class I, class II, and proximal 1q21.1 duplications were generated. We narrowed down the sizes of TAR deletions/proximal 1q21.1 duplications and class I deletions/duplications down to minimal sizes approximately 200 and 800 kb as being reported (Cooper et al., 2011; Albers et al., 2013).

Among those 36 cases, cases 1–18 each carried one deletion at 1q21.1, cases 19 and 20 had a 358-kb deletion at the proximal 1q21.1 region (TAR deletion), nine individuals (cases 21–29) bore a 1q21.1 duplication, and the remaining seven (cases 30–36) carried a proximal 1q21.1 duplication (Figures 1A,B). The breakpoints among those cases varied dramatically, but all could be mapped to one of the segmental duplication blocks (BP1–BP4). Fourteen out of 18 deletions at 1q21.1 belonged to class I deletions, which occurred between BP3 and BP4 with a size range of 1.3–2.2 Mb, while the remaining four were categorized as class II deletions with larger sizes, ranging from 3.0 to 3.9 Mb and flanked by BP1/BP2 and BP4. Only two patients carried the typical TAR deletion of ∼350 kb (Figure 1A) (Albers et al., 2013).

Both class I and class II duplications at the 1q21.1 locus were likewise detected in our cohort. Specifically, we found class I duplications among eight individuals (cases 21–26 and cases 28 and 29) and a class II duplication in one patient (case 27) who carried the largest duplication of 4.9 Mb, flanked by BP1 and BP4 (Figure 1B). Surprisingly, we identified seven individuals (cases 30–36) possessing proximal 1q21.1 duplications with a size range of 228 kb–1.1 Mb. Among them, cases 30 and 31, a mother and her daughter, carried the largest duplication of 1.1 Mb, spanning BP1 and BP3 (Figure 1B).

### Clinical Manifestations

**FIGURE 2:**
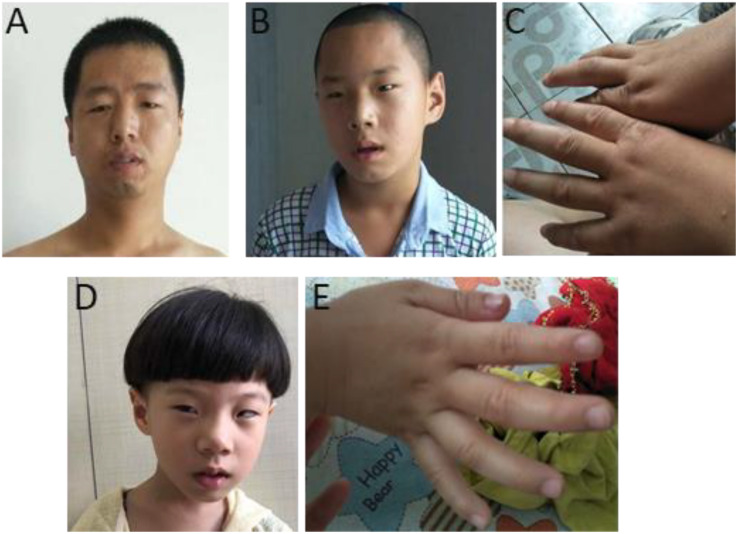
Photos of one family with 1q21.1 deletions (cases 7–9). **(A)** The father with mild dysmorphic facial features and the son with intellectual disabilities, strabismus, deformed ears **(B)**, and tapered fingers **(C)**. **(D,E)** The daughter has intellectual disabilities. Like her elder brother, she also manifested with strabismus, pointed chin and deformed ears **(D)**, and tapered fingers **(E)**.

The phenotypes for individuals with 1q21.1 deletions are summarized in Table 1. Among 18 individuals who carried either a type I or a type II deletion, nine (50%) displayed growth delay with either height percentile, weight percentile, or both lower than 95% of the same age peers within the general population. Three of 18 (17%) had a significantly small head (2nd percentile head circumference), and no individual displayed macrocephaly. Mild to moderate dysmorphic facial features were recognized in 10 patients (56%). Cognitive and/or behavioral defects appeared as another relatively common phenotype, manifesting in 10 of 18 (56%) individuals. Other clinical features identified in at least two individuals included cardiac defects (five patients), constipation (five patients), failure to thrive (FTT; three patients), gastroesophageal reflux disease (three patients), ataxia (two patients), hypotonia (two patients), and uterus malformation (two female patients). Notably, three parents of four patients either presented with unrecognizable phenotypic features (case 5, mother of case 4) or only mild symptoms (case 7, father of cases 8 and 9; case 13, mother of case 14), even though they carried identical deletions as their children did. As shown in Figure 2, the son and daughter displayed apparent dysmorphic facial anomalies and had profound IDs when compared to the father, suggesting incomplete penetrance and a variable expressivity of 1q21.1 deletion as reported by other groups (Brunetti-Pierri et al., 2008; Mefford et al., 2008; Wang et al., 2017; Chen et al., 2018). In general, no apparent distinct features were observed among the carriers of class I and class II deletions, which is consistent with previous reports (Brunetti-Pierri et al., 2008; Mefford et al., 2008).

**TABLE 1:** Molecular characterization and phenotypical features for individuals with 1q21.1 deletions.

Case	Age	Sex	Size	Growth features^a^	Head size (percentile) and facial features^b^	Cognitive and behavioral features	Cardiac anomalies	Additional clinical features	Notes
1	3 Y	M	1.8 Mb	0%; 0%	Normal; normal	No	No	FTT, delayed bone age (<2 SD of the mean), constipation	∼
2	3 Y	M	1.3 Mb	3%; 7%	Microcephaly (0%); dysmorphic facial features, possible Pierre Robin sequence	Speech and fine motor delays	No	Constipation, eczema	∼
3	5 Y	F	2.1 Mb	85%; 95%	Normal; esophoria	Speech, motor skills and cognition delays, behavioral concerns	PFO, PDA	Gastroschisis, esophagus stenosis, proteinuria, constipation	∼
4	10 M	M	3.0 Mb	74%; 64%	Normal; normal	No	Systolic heart murmur	Type-1 laryngeal cleft, ankyloglossia, GERD, dysphagia, tonsillar hypertrophy	∼
5	36 Y	F	3.0 Mb	N/A	Normal; normal	No	No	No	MOC 4
6	6 Y	M	1.2 Mb	2%; 16%	Normal; normal	Fine motor and mild gross motor delays	Ascending aorta dilatation, bicuspid valve	Laryngotracheomalacia, GERD, connective tissue disorder, hydrocele, bilateral eustachian tube dysfunction	∼
7	36 Y	M	1.3 Mb	N/A	Normal; prominent nasal bridge, mild micrognathia	No	No	No	FOC 8 and 9
8	12 Y	M	1.7 Mb	N/A	Normal; strabismus, prominent nasal bridge, deformed ears	ID	No	Tapered fingers, ataxia, abnormal eye movement	∼
9	5 Y	F	1.7 Mb	15%; 1%	Normal; strabismus, deformed ears, hypertelorism, pointed chin	ID	No	Tapered fingers, ataxia, abnormal eye movement	∼
10	11 Y	F	1.3 Mb	1%; 1%	Normal; normal	ADHD, ID, disruptive behavior disorder, anxiety	PDA	Congenital megaureter, dysfunction bladder, solitary kidney, short stature	∼
11	16 M	M	1.3 Mb	0%; 0%	Normal; dropped upper eyelids with bilateral colobomas	No	No	Hypotonia, dysphagia, laryngomalacia, glottic stenosis, FTT, inguinal hernia, GERD, phimosis	∼
12	23 Y	F	1.3 Mb	N/A	N/A	No	No	Bicornuate uterus, two preterm deliveries with one fetal demise who also carries the deletion	∼
13	23 Y	F	2.2 Mb	N/A	Normal; high-arched eyebrows	Bipolar	No	No	MOC 14
14	3 Y	F	2.2 Mb	1%; 7%	Microcephaly (<1%); high arched eyebrows, medial epicanthal folds, retrognathic jaw with protruding upper lip	Mild social and speech delays	No	Constipation, FTT	∼
15	21 M	F	1.8 Mb	27%; 74%	Normal; mild hypotelorism, large and cupped ears	ID, speech and motor delay	No	No	∼
16	21 M	F	1.5 Mb	0%; 7%	Microcephaly (1%); normal	Disruptive behavior, ID, speech delay	PFO	Hypotonia, seizure, apnea, dysphagia, constipation, tongue-tie	∼
17	16 Y	M	2.5 Mb	2%; 70%	Normal; normal	No	No	Aplasia of uterus, forearm deformity with radial hypoplasia, underdeveloped hand; clinical features suggestive of Mayer-Rokitansky syndrome	∼
18	12 Y	M	3.9 Mb	76%; 54%	Normal; low posterior hairline, synophrys, mouth with high-arched palate, dental crowding, small chin	Learning difficulties, behavior disorders	No	Long and webbing neck	∼
19	19 M	M	358 Kb	1%; 28%	Normal; normal	N/A	PFO, small PDA	Bilateral absent radii with shortened forearm, thrombocytopenia, TAR syndrome	∼
20	26 M	F	358 Kb	1%; 0%	Microcephaly (0.1%); nystagmus	Speech delay	No	Extreme prematurity (23 weeks), moderate hearing loss, adenoids hypertrophy, agenesis of the corpus callosum, hypotonia, spastic cerebral palsy, FTT	∼

*^*a*^Growth features are illustrated by height percentile followed by weight percentile; ^*b*^Head size is represented by head circumference percentile. ADHD, attention-deficit/hyperactivity disorder; FTT, failure to thrive; GERD, gastroesophageal reflux disease; ID, intellectual disabilities; PDA, patent ductus arteriosus; PFO, patent foramen ovale; MOC, mother of case; FOC, father of case; N/A, not available.*

Two patients who carried the TAR deletion displayed non-overlapping clinical features despite sharing an identical 358-kb deletion (Table 1). Patient 19 had the characteristic features of TAR syndrome—bilateral absent radii with shortened forearm and thrombocytopenia. This patient received a clinical diagnosis of TAR syndrome prior to receiving the result of the microarray test. Patient 20 was an extremely premature infant (born at 23 weeks) with agenesis of the corpus callosum, hypotonia, seizures, and mild hearing loss. It was not clear if this infant could be diagnosed with TAR syndrome because of the lack of typical clinical features. TAR syndrome was recently classified as an autosomal recessive disorder (Klopocki et al., 2007); however, the patient’s DNA sample was not available to test if she carried another pathogenic variant in trans.

We also outlined in Table 2 the clinical features of nine individuals with 1q21.1 duplications and seven with proximal 1q21.1 duplications. Contrastingly, the undergrowth phenotype found in patients with 1q21.1 deletions was mostly replaced by overgrowth in patients with 1q21.1 duplications: four out of nine patients exhibited overgrowth of either height, weight, or both in the top 2 percentile. Cognitive and behavioral issues were typical for patients with 1q21.1 duplications, identified in two-thirds of the patients (six out of nine). Autistic features were observed in 33% (three out of nine) of patients. Other common clinical phenotypes included macrocephaly/mild dysmorphic facial features (three patients) and cardiac anomalies (three patients). In addition, two male infants, one 14-month-old (case 23) and one 23-month-old (case 24), with 1q21.1 duplications exhibited hearing loss. It is worth mentioning that the patients’ ages varied from 1 month to 14 years. Certain clinical features, for instance, psychiatric problems, might not be recognizable in infants. In those nine patients, only one (case 27) carried a class II duplication of 4.9 Mb, and the remaining eight patients had class I duplications with somewhat similar sizes at around 1.3 Mb. Phenotypically, class I and class II duplications were indistinguishable as illustrated in earlier reports (Brunetti-Pierri et al., 2008; Mefford et al., 2008; Rosenfeld et al., 2012).

**TABLE 2:** Molecular characterization and phenotypical features for individuals with 1q21.1 duplications.

Case	Age	Sex	Size	Growth features^a^	Head size (percentile) & facial features^b^	Cognitive and behavioral features	Cardiac anomalies	Additional clinical features	Notes
21	6 Y	F	1.3 Mb	99%; 100%	Macrocephaly (98%); up slanting palpebral fissures, helix folded ears	Speech delay	No	Port-Wine stain on neck, fifth finger brachydactyly, sandal gap feet	
22	1 M	F	1.3 Mb	11%; 38%	Normal; normal	N/A	PDA, ASD, tricuspid regurgitations, Epstein anomaly	Tachycardia	
23	14 M	M	1.3 Mb	2%; 0%	Normal; normal	No	TOF with pulmonary atresia	Hearing loss, feeding difficulties	
24	23 M	M	1.3 Mb	24%; 92%	Normal; normal	Speech and gross motor delays	PFO	Hypotonia, bilateral metatarsus adductus, gait abnormalities, hearing loss, congenital anomaly of great vein, phimosis	
25	14 Y	M	1.2 Mb	70%; 99%	Macrocephaly (99%); flat nasal bridge	Speech and fine motor delays	No	Autism, Tourette syndrome, Sydenham chorea, hippocampal volume loss, hepatosplenomegaly, constipation, myopia, inguinal hernia, acanthosis nigricans	
26	3 Y	M	1.3 Mb	98%; 87%	Normal; normal	No	No	Imperforate anus with perineal fistula	
27	13 Y	F	4.9 Mb	11%, 61%	Macrocephaly (98%); mildly overfolded ears	Speech disorder, behavioral problem, ADHD	No	Autism, anemia	
28	8 Y	M	1.5 Mb	97%, 99%	Normal; normal	ID, speech and fine motor delays, ADHD	No	No	
29	5 Y	M	1.9 Mb	39%; 53%	Normal; normal	Behavior problem, speech and motor delays, ADHD	No	Autism	
30	25 Y	F	1.1 Mb	N/A	Normal; normal	No	No	N/A	MOC 31
31	1.5 M	F	1.1 Mb	7%; <3%	Microcephaly (0.1%); normal	No	Balanced complete atrioventricular canal with aortic arch hypoplasia, ASD, enlarged right atrium	Leftward stomach	
32	18 M	F	228 Kb	95%; 97%	Macrocephaly (100%); mild facial dysmorphism	Speech and motor skill delays	No	Encephalomalacia, mild retinopathy, keratosis pilaris on thighs, mild hypotonia	
33	24 Y	M	341 Kb	99%; 37%	Normal; brachycephalic, short forehead, triangular shaped face, micrognathia	Verbal apraxia, ID, anxiety.	ASD	Polydactyly, arachnoid cyst	
34	29 Y	F	359 Kb	N/A	N/A	Bipolar, ID	No	Epilepsy, hypoglycemia	MOC 35
35	18 M	F	359 Kb	6%; 86%	Normal; normal	No	No	Epilepsy, oropharyngeal dysphagia, vesicoureteral reflux, hydronephrosis, history of IUGR	
36	5 M	M	622 Kb	95%; >97%	Normal; normal	No	PFO, ASD	Congenital hydrocephalus, hydronephrosis, epilepsy, cortical blindness with retinal detachment, absent optic nerves	

*^*a*^Growth features are illustrated by height percentile followed by weight percentile; ^*b*^Head size is represented by head circumference percentile. ASD, atrial septal defects; ADHD, attention-deficit/hyperactivity disorder; ID, intellectual disabilities; IUGR, intrauterine growth restriction; PDA, patent ductus arteriosus; PFO, patent foramen ovale; TOF, tetralogy of Fallot; MOC, mother of case; FOC, father of case; N/A, not available.*

**FIGURE 3:**
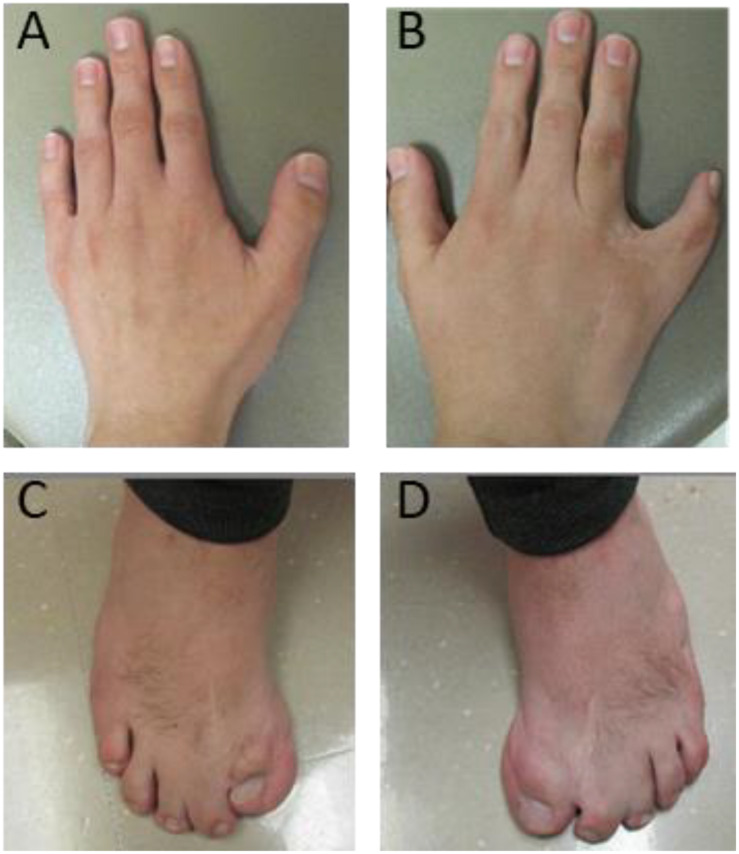
Deformed hands and feet for case 33 with a proximal 1q21.1 duplication. The patient was born with severe syndactyly and polydactyly, including six fingers on left hand **(A)**, seven fingers on right hand **(B)**, seven toes on left foot **(C)**, and six toes on right foot **(D)**. Extra fingers and toes were surgically removed. However, syndactyly is still visible on his right hand **(B)** and feet **(C,D)**.

The seven individuals with proximal 1q21.1 duplications included the two mothers (cases 30 and 34) and their daughters (cases 31 and 35). Of those two mothers, case 30 was healthy, and case 34 exhibited mild symptoms comprising of bipolar, ID, and epilepsy. Overall, the patients in this group showed more diverse clinical features. For example, the 1.5-month-old infant manifested microcephaly (case 31), and the 18-month-old showed macrocephaly (case 32). However, typical phenotypes were indeed prominent, such as cardiac defects (three patients or 43%), epilepsy (three patients), cognitive and behavioral problems (three patients), and retina defects (two patients). Case 33 was unique, a 24-year-old male with syndactyly and polydactyly. The patient was born with six fingers on his left hand, seven fingers on his right hand, seven toes on his left foot, and six toes on his right foot. The extra digits were surgically removed (Figure 3). This patient also displayed dysmorphic facial features including brachycephaly, short forehead, triangular-shaped face, micrognathia, submucosal cleft, similar to the phenotypes of OFD syndrome VI. However, both targeted sequencing of OFD syndrome-related genes (C2CD3, CPLANE1, DDX59, OFD1, and ICTN3) and whole-exome sequencing turned out to be negative (Supplementary Table S1). In addition, this patient also demonstrated ASD and arachnoid cysts. He exhibited verbal apraxia, and his latest intelligence quotient score was 41, far below average.

## Discussion

Chromosomal microarray (CMA) technologies, including SNP array and CGH array, are powerful tools for the investigation of chromosomal abnormalities. The applications of CMA technologies have led to the discovery of pathogenicity in many patients with developmental delay (Sharp et al., 2006), IDs (de Vries et al., 2005; Sagoo et al., 2009), congenital heart defects (Christiansen et al., 2004), autism (Sebat et al., 2007; Marshall et al., 2008), and schizophrenia (International Schizophrenia Consortium, 2008; Stefansson et al., 2008). The guidelines regarding how to implement those technologies and how to interpret the findings clinically have been published by the ACMG and other medical associations (Manning, 2008; Manning et al., 2010; American College of Obstetricians and Gynecologists Committee on Genetics, 2013; American College of Obstetricians and Gynecologists’ Committee on Practice Bulletins—Obstetrics et al., 2016). As a CAP-certified clinical genetic laboratory, we have been utilizing CMA technologies in patients with developmental delays, IDs, and other anomalies since 2008, particularly focusing on children and, occasionally, their parents. A frequent observation of copy number changes at 1q21.1 in patients with diverse clinical manifestations triggered our investigation into the molecular characteristics of CNVs and the genotype–phenotype correlation.

**FIGURE 4:**
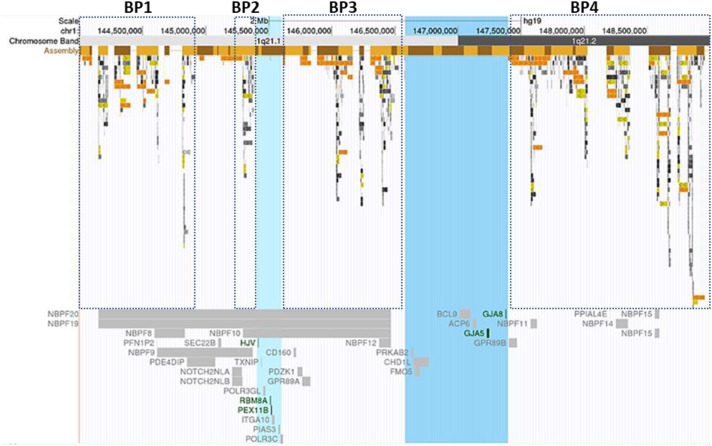
Segmental duplications and Online Mendelian Inheritance in Man (OMIM) documented genes at the 1q21.1 region. Breakpoint regions (BP1–BP4) in dotted squares were delineated according to Mefford et al. (2008). Highlighted in light blue was the minimal thrombocytopenia-absent radius deletion/proximal 1q21.1 duplication region; the blue-colored band represented the core region of class I deletions/duplications. Low copy repeats with varied sequence similarity were highlighted with different colors. Light to dark gray: 90–98%, light to dark yellow: 98–99%, and light to dark orange: >99%. The figure was generated using the University of California, Santa Cruz Genome Browser (Bailey et al., 2001, 2002). Green color genes: the molecular mechanism for the gene-associated disorders has been demonstrated. Gray color genes: the gene-related OMIM phenotype was not available.

In this study, we reported 36 cases with comprehensive, recurrent deletions and duplications at the 1q21.1 locus. To our knowledge, this is the first study that covers all four distinct types of copy number changes at this region in patients with variable symptoms. The sizes of deletions and the reciprocal duplications in the 1q21.1 region varied, ranging from 1.2 Mb (case 25) to 4.9 Mb (case 27). Nevertheless, each of those CNVs encompassed at least a minimal class I deletion/duplication of ∼800 kb and comprised seven genes (Bernier et al., 2016) (Figure 4). The breakpoints of deletions and duplications were mapped to the four breakpoint regions (BP1–BP4). Specifically, 22 out of 27 deletions/duplications at the 1q21.1 region occurred between BP3 and BP4; the remaining five took place in the middle of BP1/BP2 and BP4 (Figures 1A,B). All nine proximal deletions/duplications likewise contained the minimal 200-kb segment in the proximal 1q21.1 region defined by Klopocki et al. (2007). Two patients carried the typical 1q21.1 proximal deletion of approximately 350 kb (cases 19 and 20). In contrast, the remaining seven individuals had diverse duplications in the proximal 1q21.1 region with a size range of 228 kb–1.1 Mb. The causation of the diverse deletions and duplications in the region has been proposed by Mefford et al. (2008). As shown in Figure 4, hundreds of fragments with sizes of 10–300 kb and sequence similarities varying from 95 to 99.9% cluster in four segmental duplication regions. Low copy repeats make this region vulnerable for NAHR during meiosis and mitosis, and this could contribute to the creation of deletions or duplications in daughter cells (Mefford and Eichler, 2009).

Hundreds of copy number changes at 1q21.1 have been reported in several large cohort studies targeted for developmental delays, IDs, congenital anomalies, autism, and schizophrenia (Brunetti-Pierri et al., 2008; Mefford et al., 2008; Stefansson et al., 2008; Itsara et al., 2009; Cooper et al., 2011; Sahoo et al., 2011). Consequently, those studies and others have led to two newly accepted autosomal dominant syndromes: chromosome 1q21.1 deletion syndrome and chromosome 1q21.1 duplication syndrome (Digilio et al., 2013). Case studies and small-scale investigations focusing on the syndromes have been occasionally published in recent years (Harvard et al., 2011; Van Dijck et al., 2015; Verhagen et al., 2015; Bernier et al., 2016; Wang et al., 2017; Chen et al., 2018). Detailed information regarding deletions and duplications at the 1q21.1 locus are summarized in Supplementary Table S2. Nevertheless, both the 1q21.1 deletion syndrome and the 1q21.1 duplication syndrome are rare disorders, and additional phenotypic features are necessary to precisely define the disorders further. Particularly, there are only a few reports in connection with proximal 1q21.1 duplications (Supplementary Table S2). The molecular characterization and the clinical consequence of proximal 1q21.1 duplications remain largely unknown.

Our study adds 27 new cases and offers new insights to 1q21.1 deletion and duplication syndromes. First, common features appeared in patients with 1q21.1 deletions, such as microcephaly, undergrowth, mild to moderate dysmorphic facial features, ID, psychiatric disorders, short stature, and cardiac anomalies despite the absence of one clinical feature common among all or the majority of patients (Table 1). The observations are consistent with two seminal studies by Mefford et al. (2008) and Brunetti-Pierri et al. (2008), respectively. Moreover, typical clinical characters were recognized in patients carrying 1q21.1 duplications as well. For example, speech delay was found in two out of three patients; macrocephaly and autistic features were identified in one out of three probands. We also observed diverse clinical manifestations—three patients’ weight percentiles were 99th percentile or above, while one 14-month male had a low weight percentile close to 0% (case 23). Other less common features in patients with 1q21.1 duplications included hand/foot dysmorphism, hearing loss, hypotonia, and hepatosplenomegaly (Table 2). Second, microcephaly and macrocephaly were associated with 1q21.1 deletions and reciprocal 1q21.1 duplications, respectively, consistent with previous reports (Brunetti-Pierri et al., 2008; Mefford et al., 2008; Rosenfeld et al., 2012). It has been proposed that the HYDIN2 gene, which is a paralog of the HYDIN gene at 16q22.2 and resides at the BP3 region, is dosage-sensitive and responsible for the head sizes in patients (Brunetti-Pierri et al., 2008). However, this gene and its role in human diseases have not been curated in OMIM. DUF1220 sequences predominantly exist in neuroblastoma breakpoint family (*NBPF*) genes (Astling et al., 2017). By applying specialized bioinformatics tools for analyzing the CNVs of DUF1220 from 42 patients with either the 1q21.1 deletion or the reciprocal duplications, Dumas et al. (2012) proposed that the DUF1220 sequences in the NBPF genes were associated with brain size anomalies (Figure 4). Lastly, autism seems common in patients with 1q21.1 duplications in this study, consistent with other reports (Sebat et al., 2007; Brunetti-Pierri et al., 2008; Marshall et al., 2008). It has been proposed that DUF1220 dosage contributes to the disease severity of autism patients (Davis et al., 2014), yet the direct linkage from the DUF1220 copy numbers to the brain sizes and the severe autistic features of patients still need to be investigated extensively in the future. Of note is that no patient carrying 1q21.1 deletions displayed symptoms related to schizophrenia as being depicted earlier (International Schizophrenia Consortium, 2008; Stefansson et al., 2008), and this is probably due to the fact that most of our patients were too young (<11 years).

Thrombocytopenia-absent radius syndrome was recognized in the 1950s (Gross et al., 1956; Shaw and Oliver, 1959), yet the genetic etiology was not fully understood until recently (Klopocki et al., 2007; Albers et al., 2013). Klopocki et al. (2007) investigated 30 unrelated patients diagnosed with TAR syndrome and found that all patients carried one common deletion at the proximal 1q21.1 region with a size range of 200–500 kb. It is now believed that TAR syndrome is an autosomal recessive disease caused by mutations in the RBM8A gene, typically including a null allele and a non-coding SNP in the 5-terminal for the other allele (Albers et al., 2013). Two patients in this study carried a proximal 1q21.1 deletion. One patient (case 19) needed frequent platelet transfusions and was diagnosed as TAR syndrome before our test, while the other patient (case 20) did not display the characteristic features of TAR syndrome. It was not clear if the patient carried an additional mutation on the other allele or whether the symptoms were directly associated with the proximal 1q21.1 deletion. Alternatively, patient 20 was a carrier of a proximal 1q21.1 deletion, and the clinical features were partially or solely related to the premature delivery. It is worth noting that there are fewer patients with proximal 1q21.1 deletion in this study because TAR syndrome can be diagnosed without genetic testing (Hall et al., 1969).

Unlike proximal 1q21.1 deletions which have been associated with TAR syndrome, there are still lots of unknowns for proximal 1q21.1 duplications. By studying a large cohort of 15,767 children with ID and other congenital abnormalities as well as 8,329 health controls, Cooper et al. (2011) reported that 26 patients in the disease cohort and only one individual in the control group carried a proximal 1q21.1 duplication, a significant enrichment with an odds ratio of 13.71 (*p* = 0.0002). Another study was jointly performed by multiple institutions in the United States which identified 20 probands with proximal 1q21.1 duplications; the clinical features identified in four or more patients included facial anomalies, FTT/feeding problems, ID/developmental disabilities, clinodactyly, skeletal limb defects, and autistic features (Rosenfeld et al., 2012). Our study incorporates seven more cases with proximal 1q21 duplications into the pool of this rare disorder. Of note is that case 30 had no recognizable clinical phenotypes, but her newborn daughter (case 31) had a head circumference below 99.9% of her same-age peers, cardiac defects, and leftward stomach, suggesting incomplete penetrance of the proximal 1q21.1 duplication. Case 33, a 24-year-old male, exhibited severe dysmorphic facial characters, polydactyly, language delay, ID, and ASD. Those characteristics resemble the phenotype of one early reported case (Brunet et al., 2009). The findings suggest that proximal 1q21.1 duplications highly likely contributed to the clinical phenotypes in patients. However, whether other genetic or environmental factors are also involved requires further explorations, especially considering that compound heterozygous mutations, including a proximal 1q21.1 deletion, are essential for TAR syndrome.

Segmental duplications at the 1q21.1 locus promote unequal crossing over and compromise the genomic stability in this region (Mefford et al., 2008; Mefford and Eichler, 2009). Because of the broad applications of CMA technologies in clinical diagnoses, more and more patients with CNVs in this region have been recognized. Apart from TAR syndrome which can be recognized easily based on the typical clinical feature and dramatically reduced blood platelet counts, the other three disorders, including proximal 1q21.1 duplication and chromosome 1q21.1 deletion/duplication syndromes, are difficult to be recognized clinically owing to variable expressivity and incomplete penetrance. Consequently, diagnoses should be more or less dependent on genetic testing, such as CMA technologies or next-generation sequencing-based assays. The characterization of each disorder also poses significant challenges for genetic counseling that requires both careful clinical evaluation and accurate genetic testing.

## Conclusion

Our large cohort study in patients with developmental delays, head and facial abnormalities, cardiac defects, psychiatric issues, and other anomalies reinforced that the subchromosomal region 1q21.1 (GRCh37/hg19, chr1:144.0–149.5 Mb) was a hotspot for deletions and duplications due to the presence of hundreds of low copy repeats. In this study, we identified diverse copy number changes at the 1q21.1 locus, including class I/II deletions, class I/II duplications, proximal 1q21.1 deletions, and reciprocal 1q21.1 duplications. Deletions and duplications in the 1q21.1 region have been associated with three syndromes, including chromosome 1q21.1 deletion syndrome, chromosome 1q21.1 duplication syndrome, and TAR syndrome. Our findings provided valuable information for those rare syndromes, both on the molecular characteristics of deletions/duplication and on the phenotypic diversity. Most importantly, our study identified seven cases with recurrent duplications in the proximal 1q21.1 region, indicating that the variants highly likely contribute the clinical manifestations in patients.

We also observed incomplete penetrance and variable expressivity to be related to copy number changes at the 1q21.1 locus. The findings suggest that the diagnoses of disorders associated with CNVs at 1q21.1 require not only careful clinical evaluation but also accurate genetic tests.

## Data Availability Statement

The datasets for this article are not publicly available due to concerns regarding participant/patient anonymity. Requests to access the datasets should be directed to the corresponding author.

## Ethics Statement

The studies involving human participants were reviewed and approved by the Institutional Review Board (IRB) of the University of Oklahoma Health Sciences Center, the ethical committee of the First Hospital of Jilin University. Written informed consent to participate in this study was provided by the participants’ legal guardian/next of kin. Written informed consent was obtained from the individual(s), and minor(s)’ legal guardian/next of kin, for the publication of any potentially identifiable images or data included in this article.

## Author Contributions

HG and SL designed the study, analyzed the data, and drafted the manuscript. HP and XY participated in collecting the patients’ information and in data analysis and wrote the manuscript. YK, XW, JJ, and JY conducted the tests and performed the result interpretation. All authors contributed to the article and approved the submitted version.

## Conflict of Interest

The authors declare that the research was conducted in the absence of any commercial or financial relationships that could be construed as a potential conflict of interest.
