# Supplementary material for: Disorders Associated With Diverse, Recurrent Deletions and Duplications at 1q21.1
Source: Front Genet. 2020 Jun 23;11:577. doi: 10.3389/fgene.2020.00577 (PMC7325322; doi:10.3389/fgene.2020.00577)
Supplement: Supplementary file 1 [file Table_1.docx]

Supplementary table 1. Copy number changes identified in 36 individuals and the initial reasons for genetic testing

| Case^a^ |  | Lab ID | Age | Sex | Test | Additional test^b^ | Genome coordinates (GRCh37/hg19) | Size | Reasons for study^c^ |
| --- | --- | --- | --- | --- | --- | --- | --- | --- | --- |
| 1 |  | Mo2019-001182 | 3 Y | M | SNP | Karyotype (-) | 1q21.1q21.2(146089268_147911246)x1 | 1.8 Mb | Short stature |
| 2 |  | Mo2019-000858 | 3 Y | M | CGH | N/A | 1q21.1q21.2(146507710-147828089)x1 | 1.3 Mb | Microcephaly, Pierre Robin syndrome, speech delay |
| 3 |  | 18-0680-DB2 | 5 Y | F | SNP | N/A | 1q21.1q21.2(145,762,959-147,911,246)x1 | 2.1 Mb | DD |
| 4 |  | 18-0417-DB2 | 10 M | M | SNP | N/A | 1q21.1q21.2(144,854,574-147,806,652)x1 | 3.0 Mb | Family history of congenital malformations, deformations |
| 5 |  | 17-0365-Db2 | 36 Y | F | CGH | N/A | 1q21.1q21.2(144,854,574-147,806,652)x1 | 3.0 Mb | Two children with 1q21.1 deletion |
| 6 |  | 18-0361-Db2 | 6 Y | M | SNP | N/A | 1q21.1q21.2(146507710-147745610)x1 | 1.2 Mb | Connective tissue diseases |
| 7 |  | 17-0326-D research | 36 Y | M | CGH | N/A | 1q21.1q21.2(146,507,710-147,828,089)x1 | 1.3 Mb | Father of case 8 and 9 |
| 8 |  | 17-0328-D research | 12 Y | M | SNP | N/A | 1q21.1q21.2(146,152,553-147,825,454)x1 | 1.7 Mb | ID |
| 9 |  | 17-0329-D research | 7Y | F | SNP | N/A | 1q21.1q21.2(146,152,553-147,825,454)x1 | 1.7 Mb | ID |
| 10 |  | 15-0186-Db2 | 11 Y | F | CGH | N/A | 1q21.1q21.2(146,507,710-147,828,089)x1 | 1.3 Mb | Short stature, underweight, ID, one kidney |
| 11 |  | 14-1098-DB2 | 16 M | M | CGH | N/A | 1q21.1q21.2(146,542,843-147,828,089)x1 | 1.3 Mb | Hypoxia |
| 12 |  | 13-1140-Db2 | 23 Y | F | CGH | Karyotype (-) | 1q21.1q21.2(146,501,144-147,826,644)x1 | 1.3 Mb | Fetus with chromosomal anomalies |
| 13 |  | 11-0656-Db2 | 23 Y | F | CGH | N/A | 1q21.1(145388977-147628560)x1 | 2.2 Mb | Mother of case 14 |
| 14 |  | 11-0318-Db2 | 3 Y | F | CGH | Karyotype (-) | 1q21.1(145388977-147628560)x1 | 2.2 Mb | Failure to thrive |
| 15 |  | 11-0239-DB2 | 21 M | F | CGH | Karyotype (-) | 1q21.1(146084826-147895683)x1 | 1.8 Mb | DD, microcephaly |
| 16 |  | 11-0196-Db2 | 21 M | F | CGH | Karyotype (-) | 1q21.1(145934459-147389884)x1 | 1.5 Mb | DD, microcephaly |
| 17 |  | 10-0909-Db2 | 16 Y | M | CGH | Karyotype (-) | 1q21.1(145384335-147895683)x1 | 2.5 Mb | Absent uterus |
| 18 |  | 10-0553-Db2 | 12 Y | M | CGH | Karyotype (-) | 1q21.1(144890951-148811197)x1 | 3.9 Mb | DD |
| 19 |  | 11-0111-Db2 | 19 M | M | CGH | N/A | 1q21.1(145388977-145746649)x1 | 358 Kb | TAR syndrome |
| 20 |  | 10-0466-Db2 | 26 M | F | CGH | Karyotype (-) | 1q21.1(145388977-145746649)x1 | 358 Kb | Microcephaly |
| 21 |  | Mo2019-000746 | 6 Y | F | SNP | N/A | 1q21.1q21.2(146506691_147826789)x3 | 1.3 Mb | Macrocephaly, DD |
| 22 |  | 19-0047-DB2 | 1 M | F | SNP | N/A | 1q21.1q21.2(146,501,348-147,795,939)x3 | 1.3 Mb | Large for gestational age, Epstein anomaly |
| 23 |  | 18-0712-DB2 | 14 M | M | SNP | N/A | 1q21.1q21.2(146,501,348-147,825,548)x3 | 1.3 Mb | N/A |
| 24 |  | 18-0581-DB2 | 23 M | M | SNP | N/A | 1q21.1q21.2(146,556,146-147,825,548)x3 | 1.3 Mb | Speech delay |
| 25 |  | 17-1240-DB2 | 14 Y | M | SNP | N/A | 1q21.1q21.2(146,544,721-147,808,631)x3 | 1.2 Mb | Autism |
| 26 |  | 16-0693-Db2 | 3 Y | M | CGH | N/A | 1q21.1q21.2(146,508,774-147,825,454)x3 | 1.3 Mb | Imperforate anus |
| 27 |  | 14-0313-DB2 | 13 Y | F | CGH | N/A | 1q21.1q21.2(144,009,907-148,936,712)x3 | 4.9 Mb | Behavioral problems, speech disorder |
| 28 |  | 13-1052-DB2 | 8 Y | M | CGH | N/A | 1q21.1q21.2(146,310,267-147,824,207)x3 | 1.5 Mb | DD |
| 29 |  | 12-0028-Db2 | 5 Y | M | CGH | Karyotype (-) | 1q21.1(145750007-147904082)x3 | 1.9 Mb | N/A |
| 30 |  | 18-0464-DB2 | 25 Y | F | SNP | N/A | 1q21.1(144,617,179-145,704,639)x3 | 1.1 Mb | Family history |
| 31 |  | 18-0341-Db2 | 1.5 M | F | SNP | N/A | 1q21.1(144,617,179-145,704,639)x3 | 1.1 Mb | Atrioventricular canal defect |
| 32 |  | 18-0346-DB2 | 18 M | F | CGH | N/A | 1q21.1(145,382,362_145,610,761)x3 | 228 Kb | N/A |
| 33 |  | 18-0148-DB2 | 24 Y | M | SNP | WES (-); OFD syndrome panel (-) | 1q21.1(145,394,955-145,736,438)x3 | 341 Kb | Polydactyly |
| 34 |  | 15-1100-Db2 | 29 Y | F | CGH | Karyotype (-) | 1q21.1(145,388,355-145,747,269)x3 | 359 Kb | Daughter with chromosome anomaly |
| 35 |  | 15-0825-Db2 | 18 M | F | CGH | N/A | 1q21.1(145,388,355-145,747,269)x3 | 359 Kb | Microcephaly, failure to thrive, seizure |
| 36 |  | 11-0526-DB2 | 5 M | M | CGH | N/A | 1q21.1(145388977-146011089)x3 | 622 Kb | Hydrocephalus |

^a.^ Additional copy number changes: Case 22, 13q21.33(70,578,306-71,599,302)x3 (~ 1 Mb); Case 23, 15q21.2(50,494,513-50,903,600)x1 (~409Kb). ^b.^ “-” normal; WES, whole-exome sequencing, OFD, oral-facial-digital. ^C.^ DD developmental delay; ID intellectual disability; N/A, not available.
