# Supplementary material for: Disorders Associated With Diverse, Recurrent Deletions and Duplications at 1q21.1
Source: Front Genet. 2020 Jun 23;11:577. doi: 10.3389/fgene.2020.00577 (PMC7325322; doi:10.3389/fgene.2020.00577)
Supplement: Supplementary file 2 [file Table_2.pdf]

Supplementary table 2. Summary of 1q21.1 deletion and duplication cases in published case reports, family studies, disease cohorts and case-control studies

| 1q21.1 del.[a] | 1q21.1 dup. | 1q21.1 proximal dup. | 1q21.1 proximal del. | Note [b] | Reference |
|---|---|---|---|---|---|
| **Case report** | | | | | |
| 1 | | | | | (Alzarka et al., 2018) |
| | 1 | | | | (Benitez-Burraco et al., 2018) |
| | | | 1 | | (Bottillo et al., 2013) |
| | | | 2 | | (Brodie et al., 2019) |
| 1 | | | | | (Cadieux-Dion et al., 2018) |
| 7 | 1 | | | | (Ceylan et al., 2019) |
| 1 | | | | | (Chen et al., 2018) |
| 3 | 1 | | | | (Digilio et al., 2013) |
| 1 | | | | | (Gamba et al., 2016) |
| | | | 1 | | (Giordano et al., 2011) |
| | 1 | | | | (Gourari et al., 2018) |
| | | | 2 | | (Guastadisegni et al., 2012) |
| | 1 | | | | (Gulati et al., 2014) |
| | | | 11 | | (Houeijeh et al., 2011) |
| | | 1 | 3 | | (Liao et al., 2012) |
| | | | 1 | | (Papoulidis et al., 2014) |
| | 1 | | | | (Sun et al., 2015) |
| | | | 1 | | (Tassano et al., 2015) |
| | 1 | | | | (Xavier et al., 2018) |
| | 3 | | | | (Ji et al., 2018) |
| **Family study** | | | | | |
| 2 | | | | Neurodevelopmental features | (Basel-Vanagaite et al., 2011) |
| 6 | 2 | | | Congenital anomalies, ID | (Harvard et al., 2011) |
| | 11 | | | A family with 1q21.1 duplication | (Verhagen et al., 2015) |
| 4 | 1 | | | Families with CNVs at 1q21.1 | (Wang et al., 2017) |
| **Disease cohort** | | | | | |
| 4 | 2 | | | ID, DD, dysmorphic features | (Buse et al., 2017) |
| | | | 26 | TAR syndrome | (Boussion et al., 2020) |
| 21 (16,577) | 15 (16,577) | | | DD, abnormal head size and behavior | (Brunetti-Pierri et al., 2008) |
| 87 (54,407) | 94 (54,407) | | | CMA tests | (Gillentine et al., 2018) |
| | | | 30 | TAR syndrome | (Klopocki et al., 2007) |
| 13 (47,574) | | 20 (47,574) | 22 (47,574) | 1q21.1 Study | (Rosenfeld et al., 2012) |
| | 5 | | | Dysmorphic facial features | (Van Dijck et al., 2015). |
| | 2 (54) | | | Radial ray deficiencies | (Vergult et al., 2013) |
| | 1 (223) | 3 (223) | | Congenital heart disease | (Warburton et al., 2014) |
| 1 (1181) | 3 (1181) | | | Autism spectrum disease | (Autism Genome Project et al., 2007) |
| | 1 (14) | | | Mullerian aplasia | (Cheroki et al., 2008) |
| | 1 (30) | | | Kidney and urinary tract anomalies | (Weber et al., 2011) |
| 1 (132) | | | | Congenital anomalies and/or ID | (Jaillard et al., 2010) |
| 3 (505) | | | | Congenital heart disease | (Christiansen et al., 2004) |
| 19 | 19 | | | Phenotype characterization | (Bernier et al., 2016) |

| 1q21.1 del. | 1q21.1 dup. | 1q21.1 proximal dup. | 1q21.1 proximal del. | Note | Reference |
|---|---|---|---|---|---|
| | | | **Case-control** | | |
| | 3 (143) | | | Adolescent idiopathic scoliosis | (Buchan et al., 2014) |
| 47 (15,767) | 26 (15,767) | 25 (15,767) | 13 (15,767) | ID and various congenital anomalies | (Cooper et al., 2011) |
| 7 (5,650) | 13 (5,650) | | | ADHD cohort | (Gudmundsson et al., 2019) |
| 28 (6,006) | 12 (6,006) | | | Variable pediatric phenotype | (Mefford et al., 2008) |
| 1 (382) | | | | Schizophrenia | (Melhem et al., 2011) |
| 3 (2,436) | 8 (2,436) | | | Congenital heart disease | (Soemedi et al., 2012) |
| 1 (575) | | | | Schizophrenia | (Ikeda et al., 2010) |
| 17 (7918) | 2 (5179) | | | Schizophrenia | (Kirov et al., 2009) |
| 1 (572) | | | | Schizophrenia | (Need et al., 2009) |
| 4 (3945) | 7 (3945) | | | Schizophrenia | (Levinson et al., 2011) |
| 1 (751) | 2 (751) | | | Autism spectrum disease | (Weiss et al., 2008) |
| 1 (512) | 4 (512) | | | Nonsyndromic Tetralogy of Fallot | (Greenway et al., 2009) |
| **286** | **244** | **45** | **113** | | |

[a.] Numbers in parentheses () represent the total case numbers in the study, while deletions and duplications in the control group are excluded duo to unknown clinical features in most studies.

## References


Alzarka, B., Usala, R., Whitehead, M.T., and Ahn, S.Y. (2018). Hyponatremia: An Unusual Presentation in a Neonate With Chromosome 1q21.1 Deletion Syndrome. *Front Pediatr* 6**,** 273.

Autism Genome Project, C., Szatmari, P., Paterson, A.D., Zwaigenbaum, L., Roberts, W., Brian, J., Liu, X.Q., Vincent, J.B., Skaug, J.L., Thompson, A.P., Senman, L., Feuk, L., Qian, C., Bryson, S.E., Jones, M.B., Marshall, C.R., Scherer, S.W., Vieland, V.J., Bartlett, C., Mangin, L.V., Goedken, R., Segre, A., Pericak-Vance, M.A., Cuccaro, M.L., Gilbert, J.R., Wright, H.H., Abramson, R.K., Betancur, C., Bourgeron, T., Gillberg, C., Leboyer, M., Buxbaum, J.D., Davis, K.L., Hollander, E., Silverman, J.M., Hallmayer, J., Lotspeich, L., Sutcliffe, J.S., Haines, J.L., Folstein, S.E., Piven, J., Wassink, T.H., Sheffield, V., Geschwind, D.H., Bucan, M., Brown, W.T., Cantor, R.M., Constantino, J.N., Gilliam, T.C., Herbert, M., Lajonchere, C., Ledbetter, D.H., Lese-Martin, C., Miller, J., Nelson, S., Samango-Sprouse, C.A., Spence, S., State, M., Tanzi, R.E., Coon, H., Dawson, G., Devlin, B., Estes, A., Flodman, P., Klei, L., Mcmahon, W.M., Minshew, N., Munson, J., Korvatska, E., Rodier, P.M., Schellenberg, G.D., Smith, M., Spence, M.A., Stodgell, C., Tepper, P.G., Wijsman, E.M., Yu, C.E., Roge, B., Mantoulan, C., Wittemeyer, K., Poustka, A., Felder, B., Klauck, S.M., Schuster, C., Poustka, F., Bolte, S., Feineis-Matthews, S., Herbrecht, E., Schmotzer, G., Tsiantis, J., Papanikolaou, K., Maestrini, E., Bacchelli, E., Blasi, F., Carone, S., Toma, C., Van Engeland, H., De Jonge, M., Kemner, C., Koop, F., et al. (2007). Mapping autism risk loci using genetic linkage and chromosomal rearrangements. *Nat Genet* 39**,** 319-328.

Basel-Vanagaite, L., Goldberg-Stern, H., Mimouni-Bloch, A., Shkalim, V., Bohm, D., and Kohlhase, J. (2011). An emerging 1q21.1 deletion-associated neurodevelopmental phenotype. *J Child Neurol* 26**,** 113-116.

Benitez-Burraco, A., Barcos-Martinez, M., Espejo-Portero, I., Fernandez-Urquiza, M., Torres-Ruiz, R., Rodriguez-Perales, S., and Jimenez-Romero, M.S. (2018). Narrowing the Genetic Causes of Language Dysfunction in the 1q21.1 Microduplication Syndrome. *Front Pediatr* 6**,** 163.

Bernier, R., Steinman, K.J., Reilly, B., Wallace, A.S., Sherr, E.H., Pojman, N., Mefford, H.C., Gerdts, J., Earl, R., Hanson, E., Goin-Kochel, R.P., Berry, L., Kanne, S., Snyder, L.G., Spence, S., Ramocki, M.B., Evans, D.W., Spiro, J.E., Martin, C.L., Ledbetter, D.H., Chung, W.K., and Simons, V.I.P.C. (2016). Clinical phenotype of the recurrent 1q21.1 copy-number variant. *Genet Med* 18**,** 341-349.

Bottillo, I., Castori, M., De Bernardo, C., Fabbri, R., Grammatico, B., Preziosi, N., Scassellati, G.S., Silvestri, E., Spagnuolo, A., Laino, L., and Grammatico, P. (2013). Prenatal diagnosis and post-mortem examination in a

fetus with thrombocytopenia-absent radius (TAR) syndrome due to compound heterozygosity for a 1q21.1 microdeletion and a RBM8A hypomorphic allele: a case report. *BMC Res Notes* 6**,** 376.

Boussion, S., Escande, F., Jourdain, A.S., Smol, T., Brunelle, P., Duhamel, C., Alembik, Y., Attie-Bitach, T., Baujat, G., Bazin, A., Bonniere, M., Carassou, P., Carles, D., Devisme, L., Goizet, C., Goldenberg, A., Grotto, S., Guichet, A., Jouk, P.S., Loeuillet, L., Mechler, C., Michot, C., Pelluard, F., Putoux, A., Whalen, S., Ghoumid, J., Manouvrier-Hanu, S., and Petit, F. (2020). TAR syndrome: Clinical and molecular characterization of a cohort of 26 patients and description of novel noncoding variants of RBM8A. *Hum Mutat*.

Brodie, S.A., Rodriguez-Aulet, J.P., Giri, N., Dai, J., Steinberg, M., Waterfall, J.J., Roberson, D., Ballew, B.J., Zhou, W., Anzick, S.L., Jiang, Y., Wang, Y., Zhu, Y.J., Meltzer, P.S., Boland, J., Alter, B.P., and Savage, S.A. (2019). 1q21.1 deletion and a rare functional polymorphism in siblings with thrombocytopenia-absent radius-like phenotypes. *Cold Spring Harb Mol Case Stud* 5.

Brunetti-Pierri, N., Berg, J.S., Scaglia, F., Belmont, J., Bacino, C.A., Sahoo, T., Lalani, S.R., Graham, B., Lee, B., Shinawi, M., Shen, J., Kang, S.H., Pursley, A., Lotze, T., Kennedy, G., Lansky-Shafer, S., Weaver, C., Roeder, E.R., Grebe, T.A., Arnold, G.L., Hutchison, T., Reimschisel, T., Amato, S., Geragthy, M.T., Innis, J.W., Obersztyn, E., Nowakowska, B., Rosengren, S.S., Bader, P.I., Grange, D.K., Naqvi, S., Garnica, A.D., Bernes, S.M., Fong, C.T., Summers, A., Walters, W.D., Lupski, J.R., Stankiewicz, P., Cheung, S.W., and Patel, A. (2008). Recurrent reciprocal 1q21.1 deletions and duplications associated with microcephaly or macrocephaly and developmental and behavioral abnormalities. *Nat Genet* 40**,** 1466-1471.

Buchan, J.G., Alvarado, D.M., Haller, G., Aferol, H., Miller, N.H., Dobbs, M.B., and Gurnett, C.A. (2014). Are copy number variants associated with adolescent idiopathic scoliosis? *Clin Orthop Relat Res* 472**,** 3216-3225.

Buse, M., Cuttaia, H.C., Palazzo, D., Mazara, M.V., Lauricella, S.A., Malacarne, M., Pierluigi, M., Cavani, S., and Piccione, M. (2017). Expanding the phenotype of reciprocal 1q21.1 deletions and duplications: a case series. *Ital J Pediatr* 43**,** 61.

Cadieux-Dion, M., Safina, N.P., Engleman, K., Saunders, C., Repnikova, E., Raje, N., Canty, K., Farrow, E., Miller, N., Zellmer, L., and Thiffault, I. (2018). Novel heterozygous pathogenic variants in CHUK in a patient with AEC-like phenotype, immune deficiencies and 1q21.1 microdeletion syndrome: a case report. *BMC Med Genet* 19**,** 41.

Ceylan, A.C., Sahin, I., Erdem, H.B., Kayhan, G., Simsek-Kiper, P.O., Utine, G.E., Percin, F., Boduroglu, K., and Alikasifoglu, M. (2019). An eight-case 1q21 region series: novel aberrations and clinical variability with new features. *J Intellect Disabil Res* 63**,** 548-557.

Chen, C.P., Chang, S.Y., Chen, Y.N., Chern, S.R., Wu, P.S., Chen, S.W., Lai, S.T., Chuang, T.Y., Yang, C.W., Chen, L.F., and Wang, W. (2018). Prenatal diagnosis of a familial 1q21.1-q21.2 microdeletion in a fetus with polydactyly of left foot on prenatal ultrasound. *Taiwan J Obstet Gynecol* 57**,** 739-744.

Cheroki, C., Krepischi-Santos, A.C., Szuhai, K., Brenner, V., Kim, C.A., Otto, P.A., and Rosenberg, C. (2008). Genomic imbalances associated with mullerian aplasia. *J Med Genet* 45**,** 228-232.

Christiansen, J., Dyck, J.D., Elyas, B.G., Lilley, M., Bamforth, J.S., Hicks, M., Sprysak, K.A., Tomaszewski, R., Haase, S.M., Vicen-Wyhony, L.M., and Somerville, M.J. (2004). Chromosome 1q21.1 contiguous gene deletion is associated with congenital heart disease. *Circ Res* 94**,** 1429-1435.

Cooper, G.M., Coe, B.P., Girirajan, S., Rosenfeld, J.A., Vu, T.H., Baker, C., Williams, C., Stalker, H., Hamid, R., Hannig, V., Abdel-Hamid, H., Bader, P., Mccracken, E., Niyazov, D., Leppig, K., Thiese, H., Hummel, M., Alexander, N., Gorski, J., Kussmann, J., Shashi, V., Johnson, K., Rehder, C., Ballif, B.C., Shaffer, L.G., and Eichler, E.E. (2011). A copy number variation morbidity map of developmental delay. *Nat Genet* 43**,** 838-846.

Digilio, M.C., Bernardini, L., Consoli, F., Lepri, F.R., Giuffrida, M.G., Baban, A., Surace, C., Ferese, R., Angioni, A., Novelli, A., Marino, B., De Luca, A., and Dallapiccola, B. (2013). Congenital heart defects in recurrent reciprocal 1q21.1 deletion and duplication syndromes: rare association with pulmonary valve stenosis. *Eur J Med Genet* 56**,** 144-149.

Gamba, B.F., Zechi-Ceide, R.M., Kokitsu-Nakata, N.M., Vendramini-Pittoli, S., Rosenberg, C., Krepischi Santos, A.C., Ribeiro-Bicudo, L., and Richieri-Costa, A. (2016). Interstitial 1q21.1 Microdeletion Is Associated with Severe Skeletal Anomalies, Dysmorphic Face and Moderate Intellectual Disability. *Mol Syndromol* 7**,** 344-348.

Gillentine, M.A., Lupo, P.J., Stankiewicz, P., and Schaaf, C.P. (2018). An estimation of the prevalence of genomic disorders using chromosomal microarray data. *J Hum Genet* 63**,** 795-801.

Giordano, P., Cecinati, V., Grassi, M., Giordani, L., De Mattia, D., and Santoro, N. (2011). Langerhans cell histiocytosis in a pediatric patient with thrombocytopenia-absent radius syndrome and 1q21.1 deletion: case report and proposal of a rapid molecular diagnosis of 1q21.1 deletion. *Immunopharmacol Immunotoxicol* 33**,** 754-758.

Gourari, I., Schubert, R., and Prasad, A. (2018). 1q21.1 Duplication syndrome and epilepsy: Case report and review. *Neurol Genet* 4**,** e219.

Greenway, S.C., Pereira, A.C., Lin, J.C., Depalma, S.R., Israel, S.J., Mesquita, S.M., Ergul, E., Conta, J.H., Korn, J.M., Mccarroll, S.A., Gorham, J.M., Gabriel, S., Altshuler, D.M., Quintanilla-Dieck Mde, L., Artunduaga, M.A., Eavey, R.D., Plenge, R.M., Shadick, N.A., Weinblatt, M.E., De Jager, P.L., Hafler, D.A., Breitbart, R.E., Seidman, J.G., and Seidman, C.E. (2009). De novo copy number variants identify new genes and loci in isolated sporadic tetralogy of Fallot. *Nat Genet* 41**,** 931-935.

Guastadisegni, M.C., Roberto, R., L'abbate, A., Palumbo, O., Carella, M., Giordani, L., Cecinati, V., Giordano, P., and Storlazzi, C.T. (2012). Thrombocytopenia-absent-radius syndrome in a child showing a larger 1q21.1 deletion than the one in his healthy mother, and a significant downregulation of the commonly deleted genes. *Eur J Med Genet* 55**,** 120-123.

Gudmundsson, O.O., Walters, G.B., Ingason, A., Johansson, S., Zayats, T., Athanasiu, L., Sonderby, I.E., Gustafsson, O., Nawaz, M.S., Jonsson, G.F., Jonsson, L., Knappskog, P.M., Ingvarsdottir, E., Davidsdottir, K., Djurovic, S., Knudsen, G.P.S., Askeland, R.B., Haraldsdottir, G.S., Baldursson, G., Magnusson, P., Sigurdsson, E., Gudbjartsson, D.F., Stefansson, H., Andreassen, O.A., Haavik, J., Reichborn-Kjennerud, T., and Stefansson, K. (2019). Attention-deficit hyperactivity disorder shares copy number variant risk with schizophrenia and autism spectrum disorder. *Transl Psychiatry* 9**,** 258.

Gulati, G., Behrman, S., Khosla, V., and Murphy, V. (2014). Challenging behaviour in a patient with schizophrenia and a 1q21.1 duplication. *BMJ Case Rep* 2014.

Harvard, C., Strong, E., Mercier, E., Colnaghi, R., Alcantara, D., Chow, E., Martell, S., Tyson, C., Hrynchak, M., Mcgillivray, B., Hamilton, S., Marles, S., Mhanni, A., Dawson, A.J., Pavlidis, P., Qiao, Y., Holden, J.J., Lewis, S.M., O'driscoll, M., and Rajcan-Separovic, E. (2011). Understanding the impact of 1q21.1 copy number variant. *Orphanet J Rare Dis* 6**,** 54.

Houeijeh, A., Andrieux, J., Saugier-Veber, P., David, A., Goldenberg, A., Bonneau, D., Fouassier, M., Journel, H., Martinovic, J., Escande, F., Devisme, L., Bisiaux, S., Chaffiotte, C., Baux, M., Kerckaert, J.P., Holder-Espinasse, M., and Manouvrier-Hanu, S. (2011). Thrombocytopenia-absent radius (TAR) syndrome: a clinical genetic series of 14 further cases. impact of the associated 1q21.1 deletion on the genetic counselling. *Eur J Med Genet* 54**,** e471-477.

Ikeda, M., Aleksic, B., Kirov, G., Kinoshita, Y., Yamanouchi, Y., Kitajima, T., Kawashima, K., Okochi, T., Kishi, T., Zaharieva, I., Owen, M.J., O'donovan, M.C., Ozaki, N., and Iwata, N. (2010). Copy number variation in schizophrenia in the Japanese population. *Biol Psychiatry* 67**,** 283-286.

Jaillard, S., Drunat, S., Bendavid, C., Aboura, A., Etcheverry, A., Journel, H., Delahaye, A., Pasquier, L., Bonneau, D., Toutain, A., Burglen, L., Guichet, A., Pipiras, E., Gilbert-Dussardier, B., Benzacken, B., Martin-Coignard, D., Henry, C., David, A., Lucas, J., Mosser, J., David, V., Odent, S., Verloes, A., and Dubourg, C. (2010). Identification of gene copy number variations in patients with mental retardation using array-CGH: Novel syndromes in a large French series. *Eur J Med Genet* 53**,** 66-75.

Ji, X., Pan, Q., Wang, Y., Wu, Y., Zhou, J., Liu, A., Qiao, F., Ma, D., Hu, P., and Xu, Z. (2018). Prenatal Diagnosis of Recurrent Distal 1q21.1 Duplication in Three Fetuses With Ultrasound Anomalies. *Front Genet* 9**,** 275.

Kirov, G., Grozeva, D., Norton, N., Ivanov, D., Mantripragada, K.K., Holmans, P., International Schizophrenia, C., Wellcome Trust Case Control, C., Craddock, N., Owen, M.J., and O'donovan, M.C. (2009). Support for the involvement of large copy number variants in the pathogenesis of schizophrenia. *Hum Mol Genet* 18**,** 1497-1503.

Klopocki, E., Schulze, H., Strauss, G., Ott, C.E., Hall, J., Trotier, F., Fleischhauer, S., Greenhalgh, L., Newbury-Ecob, R.A., Neumann, L.M., Habenicht, R., Konig, R., Seemanova, E., Megarbane, A., Ropers, H.H., Ullmann, R., Horn, D., and Mundlos, S. (2007). Complex inheritance pattern resembling autosomal recessive inheritance involving a microdeletion in thrombocytopenia-absent radius syndrome. *Am J Hum Genet* 80**,** 232-240.

Levinson, D.F., Duan, J., Oh, S., Wang, K., Sanders, A.R., Shi, J., Zhang, N., Mowry, B.J., Olincy, A., Amin, F., Cloninger, C.R., Silverman, J.M., Buccola, N.G., Byerley, W.F., Black, D.W., Kendler, K.S., Freedman, R., Dudbridge, F., Pe'er, I., Hakonarson, H., Bergen, S.E., Fanous, A.H., Holmans, P.A., and Gejman, P.V. (2011). Copy number variants in schizophrenia: confirmation of five previous findings and new evidence for 3q29 microdeletions and VIPR2 duplications. *Am J Psychiatry* 168**,** 302-316.

Liao, C., Fu, F., Yi, C.X., Li, R., Yang, X., Xu, Q., and Li, D.Z. (2012). Prenatal diagnosis of an atypical 1q21.1 microdeletion and duplication associated with foetal urogenital abnormalities. *Gene* 507**,** 92-94.

Mefford, H.C., Sharp, A.J., Baker, C., Itsara, A., Jiang, Z., Buysse, K., Huang, S., Maloney, V.K., Crolla, J.A., Baralle, D., Collins, A., Mercer, C., Norga, K., De Ravel, T., Devriendt, K., Bongers, E.M., De Leeuw, N., Reardon, W., Gimelli, S., Bena, F., Hennekam, R.C., Male, A., Gaunt, L., Clayton-Smith, J., Simonic, I., Park, S.M., Mehta, S.G., Nik-Zainal, S., Woods, C.G., Firth, H.V., Parkin, G., Fichera, M., Reitano, S., Lo Giudice, M., Li, K.E., Casuga, I., Broomer, A., Conrad, B., Schwerzmann, M., Raber, L., Gallati, S., Striano, P., Coppola, A., Tolmie, J.L., Tobias, E.S., Lilley, C., Armengol, L., Spysschaert, Y., Verloo, P., De Coene, A., Goossens, L., Mortier, G., Speleman, F., Van Binsbergen, E., Nelen, M.R., Hochstenbach, R., Poot, M., Gallagher, L., Gill, M., Mcclellan, J., King, M.C., Regan, R., Skinner, C., Stevenson, R.E., Antonarakis, S.E., Chen, C., Estivill, X., Menten, B., Gimelli, G., Gribble, S., Schwartz, S., Sutcliffe, J.S., Walsh, T., Knight, S.J., Sebat, J., Romano, C., Schwartz, C.E., Veltman, J.A., De Vries, B.B., Vermeesch, J.R., Barber, J.C., Willatt, L., Tassabehji, M., and Eichler, E.E. (2008). Recurrent rearrangements of chromosome 1q21.1 and variable pediatric phenotypes. *N Engl J Med* 359**,** 1685-1699.

Melhem, N., Middleton, F., Mcfadden, K., Klei, L., Faraone, S.V., Vinogradov, S., Tiobech, J., Yano, V., Kuartei, S., Roeder, K., Byerley, W., Devlin, B., and Myles-Worsley, M. (2011). Copy number variants for schizophrenia and related psychotic disorders in Oceanic Palau: risk and transmission in extended pedigrees. *Biol Psychiatry* 70**,** 1115-1121.

Need, A.C., Ge, D., Weale, M.E., Maia, J., Feng, S., Heinzen, E.L., Shianna, K.V., Yoon, W., Kasperaviciute, D., Gennarelli, M., Strittmatter, W.J., Bonvicini, C., Rossi, G., Jayathilake, K., Cola, P.A., Mcevoy, J.P., Keefe, R.S., Fisher, E.M., St Jean, P.L., Giegling, I., Hartmann, A.M., Moller, H.J., Ruppert, A., Fraser, G., Crombie, C., Middleton, L.T., St Clair, D., Roses, A.D., Muglia, P., Francks, C., Rujescu, D., Meltzer, H.Y., and Goldstein, D.B. (2009). A genome-wide investigation of SNPs and CNVs in schizophrenia. *PLoS Genet* 5**,** e1000373.

Papoulidis, I., Oikonomidou, E., Orru, S., Siomou, E., Kontodiou, M., Eleftheriades, M., Bacoulas, V., Cigudosa, J.C., Suela, J., Thomaidis, L., and Manolakos, E. (2014). Prenatal detection of TAR syndrome in a fetus with compound inheritance of an RBM8A SNP and a 334kb deletion: a case report. *Mol Med Rep* 9**,** 163-165.

Rosenfeld, J.A., Traylor, R.N., Schaefer, G.B., Mcpherson, E.W., Ballif, B.C., Klopocki, E., Mundlos, S., Shaffer, L.G., Aylsworth, A.S., and Q21.1 Study, G. (2012). Proximal microdeletions and microduplications of 1q21.1 contribute to variable abnormal phenotypes. *Eur J Hum Genet* 20**,** 754-761.

Soemedi, R., Topf, A., Wilson, I.J., Darlay, R., Rahman, T., Glen, E., Hall, D., Huang, N., Bentham, J., Bhattacharya, S., Cosgrove, C., Brook, J.D., Granados-Riveron, J., Setchfield, K., Bu'lock, F., Thornborough, C., Devriendt, K., Breckpot, J., Hofbeck, M., Lathrop, M., Rauch, A., Blue, G.M., Winlaw, D.S., Hurles, M., Santibanez-Koref, M., Cordell, H.J., Goodship, J.A., and Keavney, B.D. (2012). Phenotype-specific effect of chromosome 1q21.1 rearrangements and GJA5 duplications in 2436 congenital heart disease patients and 6760 controls. *Hum Mol Genet* 21**,** 1513-1520.

Sun, G., Tan, Z., Fan, L., Wang, J., Yang, Y., and Zhang, W. (2015). 1q21.1 microduplication in a patient with mental impairment and congenital heart defect. *Mol Med Rep* 12**,** 5655-5658.

Tassano, E., Gimelli, S., Divizia, M.T., Lerone, M., Vaccari, C., Puliti, A., and Gimelli, G. (2015). Thrombocytopenia-absent radius (TAR) syndrome due to compound inheritance for a 1q21.1 microdeletion and a low-frequency noncoding RBM8A SNP: a new familial case. *Mol Cytogenet* 8**,** 87.

Van Dijck, A., Van Der Werf, I.M., Reyniers, E., Scheers, S., Azage, M., Siefkas, K., Van Der Aa, N., Lacroix, A., Rosenfeld, J., Argiropoulos, B., Davis, K., Innes, A.M., Mefford, H.C., Mortier, G., Meuwissen, M., and Kooy, R.F. (2015). Five patients with a chromosome 1q21.1 triplication show macrocephaly, increased weight and facial similarities. *Eur J Med Genet* 58**,** 503-508.

Vergult, S., Hoogeboom, A.J., Bijlsma, E.K., Sante, T., Klopocki, E., De Wilde, B., Jongmans, M., Thiel, C., Verheij, J.B., Perez-Aytes, A., Van Esch, H., Kuechler, A., Barge-Schaapveld, D.Q., Sznajer, Y., Mortier, G., and Menten, B. (2013). Complex genetics of radial ray deficiencies: screening of a cohort of 54 patients. *Genet Med* 15**,** 195-202.

Verhagen, J.M., De Leeuw, N., Papatsonis, D.N., Grijseels, E.W., De Krijger, R.R., and Wessels, M.W. (2015). Phenotypic Variability Associated with a Large Recurrent 1q21.1 Microduplication in a Three-Generation Family. *Mol Syndromol* 6**,** 71-76.

Wang, H.D., Liu, L., Wu, D., Li, T., Cui, C.Y., Zhang, L.Z., and Wang, C.Z. (2017). Clinical and molecular cytogenetic analyses of four families with 1q21.1 microdeletion or microduplication. *J Gene Med* 19.

Warburton, D., Ronemus, M., Kline, J., Jobanputra, V., Williams, I., Anyane-Yeboa, K., Chung, W., Yu, L., Wong, N., Awad, D., Yu, C.Y., Leotta, A., Kendall, J., Yamrom, B., Lee, Y.H., Wigler, M., and Levy, D. (2014). The

contribution of de novo and rare inherited copy number changes to congenital heart disease in an unselected sample of children with conotruncal defects or hypoplastic left heart disease. *Hum Genet* 133**,** 11-27.
Weber, S., Landwehr, C., Renkert, M., Hoischen, A., Wuhl, E., Denecke, J., Radlwimmer, B., Haffner, D., Schaefer, F., and Weber, R.G. (2011). Mapping candidate regions and genes for congenital anomalies of the kidneys and urinary tract (CAKUT) by array-based comparative genomic hybridization. *Nephrol Dial Transplant* 26**,** 136-143.
Weiss, L.A., Shen, Y., Korn, J.M., Arking, D.E., Miller, D.T., Fossdal, R., Saemundsen, E., Stefansson, H., Ferreira, M.A., Green, T., Platt, O.S., Ruderfer, D.M., Walsh, C.A., Altshuler, D., Chakravarti, A., Tanzi, R.E., Stefansson, K., Santangelo, S.L., Gusella, J.F., Sklar, P., Wu, B.L., Daly, M.J., and Autism, C. (2008). Association between microdeletion and microduplication at 16p11.2 and autism. *N Engl J Med* 358**,** 667-675.
Xavier, J., Zhou, B., Bilan, F., Zhang, X., Gilbert-Dussardier, B., Viaux-Savelon, S., Pattni, R., Ho, S.S., Cohen, D., Levinson, D.F., Urban, A.E., and Laurent-Levinson, C. (2018). 1q21.1 microduplication: large verbal-nonverbal performance discrepancy and ddPCR assays of HYDIN/HYDIN2 copy number. *NPJ Genom Med* 3**,** 24.
